# Statistical Modeling Applied to Deformation-Relaxation Processes in a Composite Biopolymer Network Induced by Magnetic Field

**DOI:** 10.1371/journal.pone.0169866

**Published:** 2017-01-12

**Authors:** Javier Tarrío-Saavedra, Cécilia Galindo González, Salvador Naya, Jorge López-Beceiro, Alain Ponton

**Affiliations:** 1 Department of Mathematics, EPS. University of A Coruña. Avda. Mendizábal s/n. Ferrol. Spain; 2 Matière et Systèmes Complexes CNRS, UMR 7057, Université Paris Diderot-Paris 7 Bâtiment Condorcet Case 7056, Paris; 3 Department of Industrial Engineering II, EPS. University of A Coruña. Avda. Mendizábal s/n. Ferrol. Spain; The Ohio State University, UNITED STATES

## Abstract

This study investigated a methodology based on image processing and statistics to characterize and model the deformation upon controlled and uniform magnetic field and the relaxation under zero field of droplets observed in aqueous solutions of sodium alginate incorporating magnetic maghemite nanoparticles stabilized by adsorption of citrate ions. The changes of droplet geometry were statistically analyzed using a new approach based on the data obtained from optical microscopy, image processing, nonlinear regression, evolutionary optimization, analysis of variance and resampling. Image enhancement and then image segmentation (Gaussian mixture modeling) processes were applied to extract features with reliable information of droplets dimensions from optical micrographs. The droplets deformation and relaxation trends were accurately adjusted by the Kohlrausch-Williams-Watts (KWW) function and a mean relaxation time was obtained by fitting the time evolution of geometry parameters. It was found to be proportional to the initial radius of the spherical droplets and was associated to interfacial tension.

## Introduction

Nanocomposite materials composed of a polymer network in the presence of particles is nowadays a subject of intensive research due to the possibility to modulate their mechanical [1], optical, thermal [1–4], sound, magnetic, electric properties. Indeed these complex materials are potentially attractive to the development of many technologies such as electronics, optics, sensors, actuators, drug delivery and many other biotechnological areas.

Nanoparticles of different nature (gold, silver, iron, cobalt, nickel, copper, alloys, metal-derived quantum dots) can be incorporated into polymer network [5].

A special attention has been paid to magnetic nanoparticles to elaborate novel magnetoresponsive composites whose physical properties can be controlled by applying external magnetic field. In this case macroscopic behaviour is impacted by magnetic induced chain like or clustering structures. Thus, the study of microstructure is of decisive importance in order to get a better knowledge of these magnetoresponsive composites [6].

In this work, we propose a new methodology to study the deformation of magnetic field induced droplets and their relaxation when the magnetic field is switched off in an aqueous solution of biopolymer. For this purpose image enhancement techniques and an image segmentation procedure were applied to obtain data on size and shape changes of droplets. Then characteristic time of deformation and relaxation processes was obtained by a nonlinear parametric regression model as proposed by Kohlrausch, Willians and Watts (KKW) [7].

The experimental conditions are presented in the second section. The image processing techniques applied in the present study are introduced in the third section. In the fourth, the design of experiments is described while in the fifth one the results are shown and discussed before final conclusions in the sixth section.

## Experimental

### Materials

The biopolymer was a polysaccharide extracted from marine brown algae, the alginic acid (Alginate). It is a linear copolymer containing β-1,4-D-mannuronate (M) and α-1,4-L-guluronate (G) units in various proportions and sequences depending on the season, age and the part of the plant used for extraction. With one carboxylate group in each M or G unit, alginate is a highly negatively charged polyelectrolyte at neutral or basic pH. Dissolving in water, the sodium ions and the carboxylate groups of the alginate are ionized to form the alginate polyelectrolyte and to deliver sodium ions in the medium.

The used ferrofluid was an aqueous solution of ferromagnetic nanoparticles of maghemite (γ-Fe_2_O_3_) prepared by alkaline coprecipitation of ferric and ferrous salts in water [8]. Concentrated ammonium hydroxide is added to an acidic solution of iron (II) chloride and iron (III) chloride leading to the precipitation of anionic magnetite (Fe_3_O_4_) particles. After washing with distilled water, they are stirred in nitric acid, oxidized into maghemite (γ-Fe_2_O_3_) by a boiling solution of ferric nitrate and washed with acetone. These nanoparticles coated with hydroxo ligands are spontaneously soluble in aqueous acidic solutions at pH ∼2 with NO_3_^−^ counterions. Firstly in water, the surface can be coated with citrate ligands in order to obtain dispersions at pH ∼ 7. This coating is obtained by adding trisodium citrate to the native acidic ferrofluid ([Cit]/[iron] = 0.1), which is heated at 90°C during 10 minutes. After several washings with water/acetone mixtures, a stable citrated ferrofluid with pH close to 7 is obtained.

The magnetic properties are determined by magnetization M measurements on stable dispersions under an applied magnetic field H, ranging between 0 and 800 kA/m, at room temperature with a home-made vibrating magnetometer. High field determination of M_HF_ in the saturation region allows us to determine the saturation magnetization M_S_ of the nanoparticles of volume fraction Φ as M_S_ = M_HF_/Φ. In all samples here, the size distribution of the nanoparticles can be described by a lognormal distribution of diameters with the same median diameter d_0_ ∼ 7 nm (with ln d_0_ = <ln d>) and a polydispersity index σ between 0.3 and 0.4 with a saturation magnetization M_S_ = 310 ± 25 kA/m.

### Sample preparation

A solution of sodium alginate (concentration C_alg_ = 18 g.L^-1^) was first prepared by mixing sodium alginate powder in distilled water with mechanical stirring at a speed of 400 rpm during 18 hours at room temperature.

The mixture was then obtained by introducing the ferrofluid (volume fraction of nanoparticles (Φ_NP_ = 1%) in the previous aqueous sodium alginate solution.

### Characterization methods

**Fig 1:**
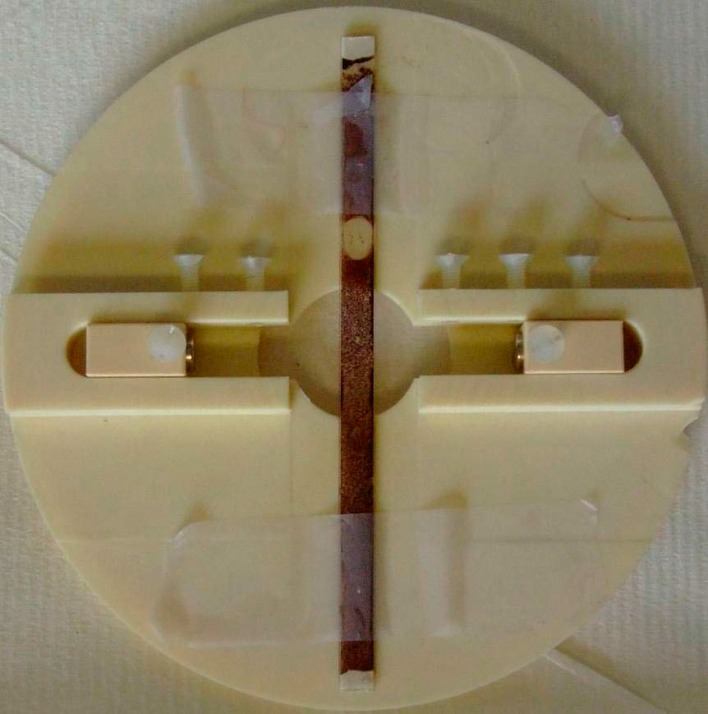
Home-made device for optical observation under application of magnetic field.

Optical microscopy observation was carried out using a laboratory made special device allowing the application of a magnetic field during the observations. Two moving permanent magnets were placed in slots of a non-magnetic circular plate (Fig 1). A rectangular capillary filled with sodium alginate solutions incorporating citrated ferrofluids was placed at the middle of the plate perpendicularly to the direction of the magnetic field. The capillary was closed on both ends by modeling clay to avoid evaporation. The observation was performed by a Nikon microscope with a magnification objective of 10x at one value of magnetic field strength (3.2 kA m^-1^). The magnetic field was applied for 5 minutes before recording images analyzed by freeware software.

### Image processing and features extraction

**Fig 2:**
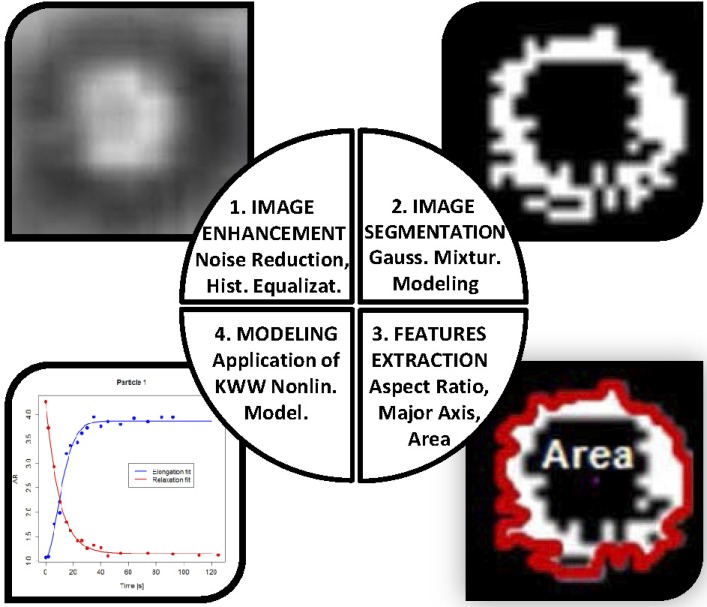
Proposed methodology scheme for studying the relaxation processes of magnetic droplets under the application of a weak magnetic field.

The application of image processing analysis was necessary to extract the droplets geometrical information from the optical micrographs. Fig 2 shows the scheme of the proposed image processing procedure that was previously used in material classification and identification tasks such as wood species [9]. This procedure provides an alternative way to collect information about relaxation processes in magnetic droplets within polymeric solutions. Image analysis application allows to extract a vector of representative features related to the geometry of the objects in the image such as diameter, area, distance, circularity, rectangularity, position, etc. [9]. ImageJ [10] and R software (through biOps library) [11] free software programs have been used to implement the enhancement and segmentation tasks (Fig 2). It is also important to note that theese processes were optimized for each one of the studied droplets.

### Image enhancement

**Fig 3:**
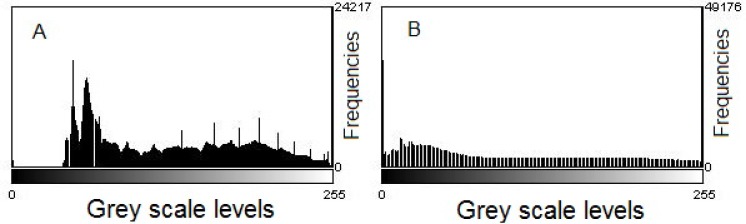
Histogram equalization procedure applied to a droplet micrograph. A: intensity histogram before the equalization process. B: intensity histogram after the application of histogram equalization process.

The obtained micrographs require the application of a set of image enhancement techniques in order to remove their noise and enhance their contrast. Noise is the random information variation of color and brightness and it may be attenuated applying some enhancement tools named filters, such as the well-known median filter [12]. In addition, the histogram equalization technique was used to improve the image contrast, i.e. a uniform intensity histogram is obtained, by applying a monotonic and nonlinear mapping (see Fig 3)

### Image segmentation

The image segmentation is a process for splitting a digital image into several groups of pixels, in this case two (black and white), so that the geometry of the droplets is clearly observed. The segmentation procedure is composed of three main steps. Estimating the brightness limit value that distinguishes objects (in this case magnetic droplets) from the background in the thresholding task, to identify the points where the brightness abruptly changes by edge detection (e.g. using Sobel operator [9]) and, finally, obtain continuous edges by applying a dilation process.

**Fig 4:**
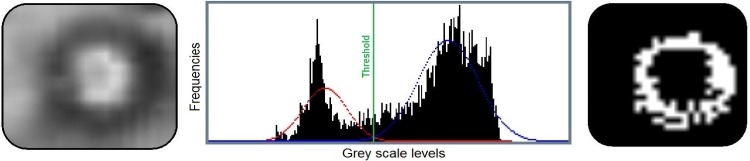
Gaussian Mixture Modeling process of segmentation applied to a droplet micrograph. Left panel: enhanced micrograph. Center: intensity histogram where two Gaussian distribution are fitted, in blue and red. Right: resulting segmented micrographs.

**Fig 5:**
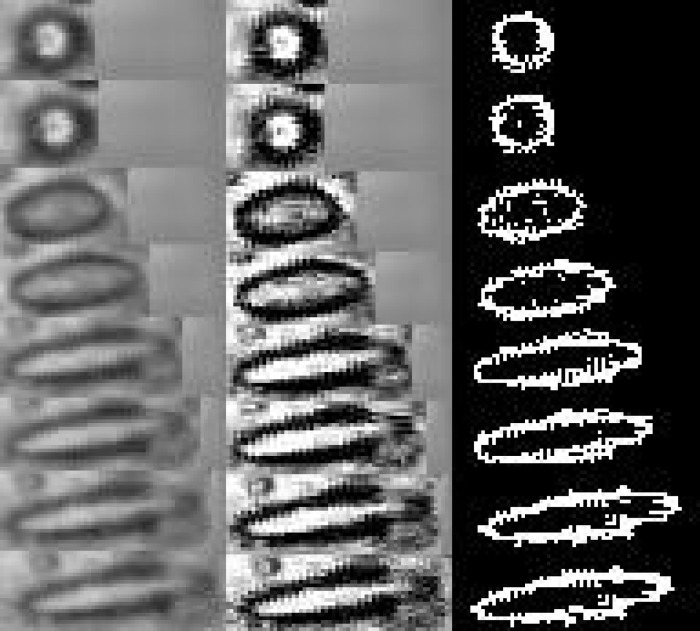
Image processing sequence corresponding to a studied droplet under the applied magnetic field.

In the present paper, the Gaussian Mixture Modeling algorithm was used for obtaining the threshold value used in the segmentation process [13]. This algorithm separates the histogram of an image into two classes by calculating the image threshold as the intersection of these two Gaussian distributions, as shown in Fig 4. This thresholding technique allows to distinguish the droplets from the background by including (in addition to threshold estimation) a grey-scale binning process and an image inversion step (see Fig 5).

Fig 5 shows the image processing sequence corresponding to a studied droplet at the times 0, 2, 6, 10, 15, 18, 23 and 26 s of application of a low magnetic field: the first column represents the original images obtained using an optical microscope, the second one shows the images after the enhancement process and the third one corresponds to the result of the segmentation process.

### Features extraction

After the segmentation process we have obtained connected objects, in this case they represent the magnetic droplets. Three characteristics related to the geometry of the droplets are extracted from the segmented images: (i) major diameter (major) corresponding to an ellipse that fit each droplet, measured in micrometer, μm, (ii) droplet area calculated by numerical methods and measured using squared micrometers, μm^2^, (iii) aspect ratio (AR), the ratio between the major and the minor diameter of the ellipse fitted to each droplet. The goal is to obtain representative features of droplets geometry, that may summarize their shape and size of droplets, in order to monitor the changes of droplet geometry depending on time during the application of magnetic field, and then when the magnetic field is switched off.

## Design of Experiments and Data Collection

**Fig 6:**
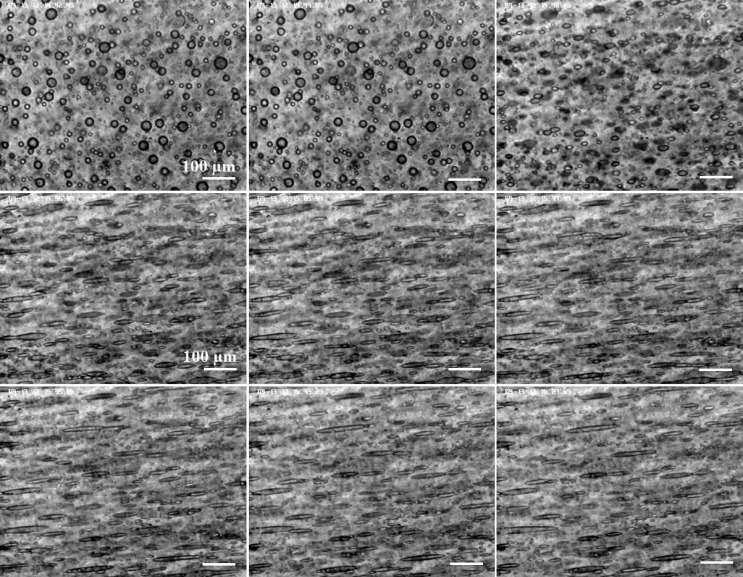
From left to right and from top to bottom, deformation of droplets under a constant value of magnetic field (3.2 kA/m) at different times 0, 6, 15, 23, 35, 45, 54, 92 and 106 s. The scale is the same for all the micrographs.

**Fig 7:**
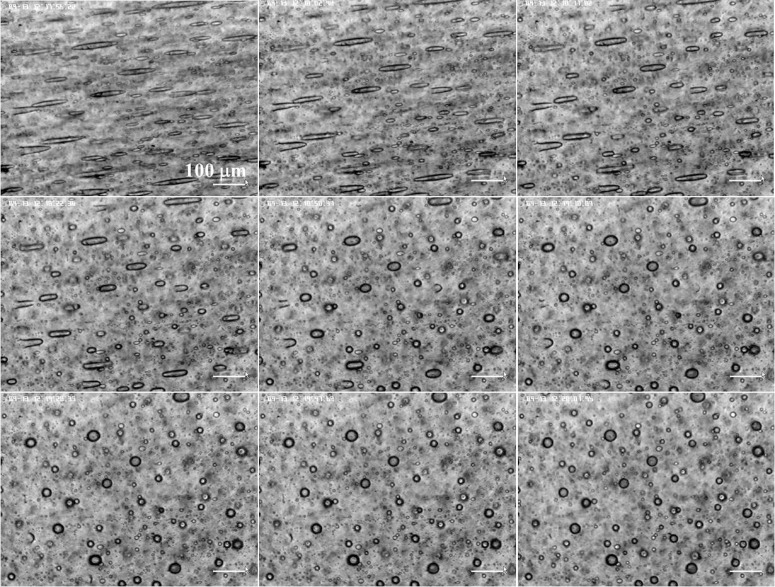
From left to right and from top to bottom, relaxation of droplets when the magnetic field is switched off at different times, at 0, 6, 15, 26, 54, 74, 92, 111 and 125 s. The scale is the same for all the micrographs.

**Fig 8:**
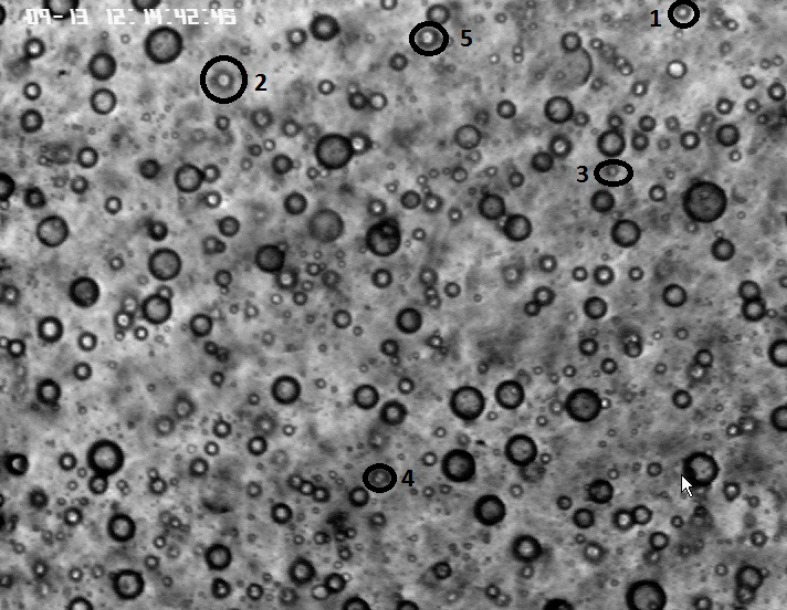
Magnetic droplets selected in the present study where neither disaggregation nor aggregation takes place.

The deformation of droplets under application of a constant value (3.2 kA m^-1^) of magnetic field and their relaxation under zero magnetic field are illustrated on Figs 6 and 7, respectively. It should be noted that droplets tend to interact with each other changing their geometrical dimensions. Droplets aggregation or disaggregation can be observed in addition to pure magnetic deformation/relaxation of individual droplets leading to the absence of mass conservation. Consequently, in order to study only the geometrical changes due to magnetic field application, 5 different droplets, of different sizes, that do not interact with the surrounding droplets were finally chosen from images. Taking into account that the most droplets interact with the others, changing their size and shape, the chosen 5 droplets is a representative sample of the overall number of droplets where there is no aggregation, disaggregation or other interaction with the surrounding area (Fig 8).

As mentioned in section 3, three different geometrical parameters were measured: area, AR, and major diameter of the ellipse that fits each droplet. In addition, we separated the measures corresponding to elongation and dimension recovery processes for each droplet. Finally, the droplet dimensions corresponding to each time were calculated attending to three variables defined as factors, applying the image analysis techniques mentioned in the next section. These three factors are the studied “droplet” (with five levels, each one corresponding to each droplet), the selected “geometric measure” (at three levels: area, AR and major diameter) and the “change of dimension process” suffered by each droplet (elongation or dimension recovery).

We want to test if the droplet geometrical changes depend on the “droplet”, “geometric measure”, and “change of dimension process” (are the magnetic induced geometric changes reversible?). Therefore, it is necessary to apply statistical design of experiments (DOE) techniques as analysis of variance (ANOVA), using F and Tukey tests [14, 15], and even multivariate analysis of variance (MANOVA). These procedures were successfully implemented in a wide range of applications [16–20].

Statistical DoE are usually performed for testing which factors or quantitative variables affect to the response variable. This response variable is a continuous variable or a vector composed of several ones. In the case of univariate response, the ANOVA is implemented, using F test to statistically infer if at least one level of the factor produces significant changes in the continuous response [14]. The Tukey test is also used to perform pairwise comparisons, i. e. to discern which factor levels produces those significant differences in the response [15]. Otherwise, concerning the multivariate response, the multivariate analysis of variance (MANOVA) with Pillai test [21, 22] was applied.

Therefore, two different models of analysis of variance, characterized by the type of response, were performed for testing the impact of each studied factor on the geometry of droplets (and thus on their relaxation processes).

On the one hand, in the MANOVA model, the response is multivariate and corresponds to the KWW nonlinear function parameters (a, b, λ_c_, β) vector [23]. This vector represents a sequence of features that summarize the information of each dimensional curve, where the AR, major diameter and area values are plotted versus the time of magnetic field application. Thus, whether differences in features vectors are statistically detected, we can infer that there will be differences in the compared geometrical curves. The MANOVA is developed, applying the Pillai test [21, 22] for checking the influence of both the multivariate response (a, b, λ_c_, β) KWW parameters vector [23]) and three factors: (i) “Droplet”, at five levels named: 1, 2, 3, 4 and 5; (ii) Geometric measure or “magnitude”, at three levels: area, AR and major diameter; (iii) change of dimension process” at two levels: droplet elongation and dimension recovery.

On the other hand, the second analysis of variance model is focused only on the characteristic relaxation times (λ_c_) obtained from each estimated KWW nonlinear equation. The aim is to focus the analysis only in characteristic relaxation time estimation, the most informative parameter of KWW model. Thus, an ANOVA model is performed to analyse the univariate response (the response variable is the λ_c_ obtained by the application of KWW model) and the same three factors as above for MANOVA:

## Results and Discussion

**Fig 9:**
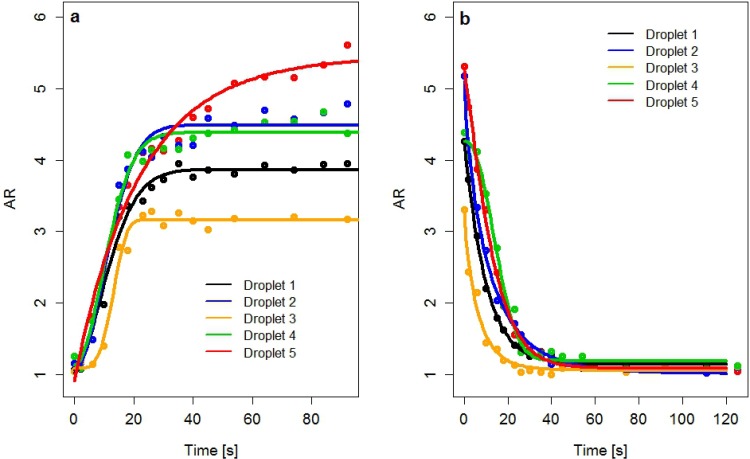
Time dependence of the AR of the 5 droplets under magnetic field (a) and when the magnetic field is switched off (b). The solid lines correspond to the fitting of experimental data by KWW nonlinear model, using the DE Algorithm.

The time dependence of AR for the 5 droplets under applied magnetic field (deformation) and when the magnetic field is switched off (relaxation) is presented on Fig 9A and 9B respectively. Similar behaviours were observed for major diameter and area of droplets. Then, experimental data were fitted by the KWW nonlinear regression model corresponding to each different “droplet” and “change of dimension process”, AR (t) or Major diameter (t) or Area (t) = a + b exp(-(t/λ_c_)^b^] where a is the initial value of the measured geometrical dimension, b is the equivalent to the instantaneous modulus in the case of viscoelastic properties, β is related to the width of the distribution of relaxation time and λ_c_ currently corresponds to a characteristic relaxation time. The value β = 1 corresponds to a monoexponential relaxation process with an unique relaxation time.

Optimum fittings were obtained by applying the Differential Evolution evolutionary global optimization algorithm (DE), through the DEoptim function of the R DEoptim package [24, 25]. The KWW parameters (a, b, λ_c_, β) obtained from fittings corresponding to the 3 geometrical indexes of the droplets are given in Table 1.

**Table 1:** KWW model parameters and R^2^ coefficients obtained applying DE optimization algorithm for each combination of the three factors. The unit of a depends on the geometric variable (area, major diameter, aspect ratio).

Elongation process					Dimension recovery process						
*a* [μm^2^] or [μm] or []	*b*	*λ*_*c*_ [s]	*β*	R^2^	*a* [μm^2^] or [μm] or []	*b*	*λ*_*c*_ [s]	*β*	R^2^	Geom. Variable	Droplet
176.1	68.13	13.22	1.31	0.90	281.9	-114.8	10.16	1.22	0.97	Area	1
319.1	320.1	16.22	2.24	1.00	641.5	-330.2	13.15	1.68	1.00	Area	
112.5	138.8	7.980	1.13	0.98	232.2	-121.8	4.560	2.04	0.94	Area	3
285.2	203.7	14.59	2.02	0.96	492.7	-195.7	12.49	4.95	0.98	Area	4
322.1	333.7	13.12	1.29	0.99	675.3	-428.4	9.520	1.38	0.98	Area	5
1.15	3.126	9.873	1.04	0.99	3.87	-2.837	13.930	1.69	0.89	AR	1
1.02	4.081	11.02	0.82	0.98	4.49	-3.417	14.121	1.91	0.99	AR	2
1.06	2.197	6.325	0.89	0.98	3.16	-2.072	14.217	4.10	0.98	AR	3
1.20	3.068	17.34	2.30	0.98	4.38	-3.244	13.515	1.94	0.99	AR	4
1.09	4.145	13.23	1.22	0.99	5.44	-4.528	22.019	0.984	1.00	AR	5
16.00	20.51	11.43	1.09	0.97	37.21	-22.21	12.62	1.58	1.00	Major	1
20.64	43.25	13.85	1.14	0.99	60.49	-39.65	13.68	1.86	0.99	Major	2
12.35	19.83	7.150	1.00	0.99	30.59	-17.70	11.61	12.0	1.00	Major	3
20.83	30.66	16.87	2.16	0.99	52.13	-30.20	12.75	2.87	1.00	Major	4
21.20	44.95	13.66	1.25	1.00	67.13	-50.19	14.84	1.06	1.00	Major	5

The good fittings of experimental data are attested by the values of the determination coefficients (R^2^) [14]. Whatever the geometrical parameter (area, AR or major diameter), it could be observed that the characteristic relaxation time λ_c_ is in the same order of magnitude for the deformation and relaxation of droplets indicating a reversibility of the process. There is only one exception for droplet 3 that could be explained by its smaller size. Moreover in most case the exponent β is close to one corresponding to a process with a single relaxation time [23].

To obtain a statistical evidence of the reversibility and similar trends for droplets, MANOVA and ANOVA tests were applied. Firstly, a MANOVA model is performed in order to analyse the multivariate response or vector of characteristics that summarize the information of each curve. Table 2 shows the obtained results. Observing the p-values corresponding to each factor, and defining a standard signification level equal to 0.05, we can conclude that the “magnitude” and “change of dimension process” are significant factors (p-values < 0.05); their variations produce significant changes in the response, the vector of KWW parameters and, therefore, in the geometric dimension curves related to deformation/relaxation processes involved. Then, the dimension curves are different depending on the measured “magnitude” and the studied “process” (deformation or relaxation, elongation or dimension recovery). In addition, Table 2 shows that the response, the dimension curves and thus the relaxation processes do not depend on the droplets in alginate solution. This result is related to the fact that almost all the particles present a similar initial size.

**Table 2:** MANOVA table for (a, b, λ_c_ β) response: Df are the degrees of freedom, Pillai represent the value of the statistic for each factor, approx. F is the approximate value for F statistic while p-value is the probability of obtaining a sample farthest from null hypothesis (no relation between factor and multiple response) than the obtained using the present sample. If p-value < 0.05, we suppose that the factor is significant regarding to response.

	Df	Pillai	approx. F	p-value
**Magnitude**	2	0.95638	45.821	0.0005084
**Droplet**	4	0.89450	15.842	0.0899211
**Process**	1	0.40186	31.913	0.0365152
**Residuals**	22			

According to the MANOVA results, the geometrical changes of droplets are different depending on “magnitude” and “process”. But testing if there are differences in the characteristic time of the process is not performed yet. This is the more informative parameter, and the index of the droplets deformation/relaxation reversibility in terms of time. Thus, for completing the analysis of variance, an ANOVA table was then performed using only the characteristic time *λ*_*c*_ as response variable (Table 3). The p-values corresponding to “magnitude” and “process” factors indicate that these factors are not significant in the estimated characteristic time (p-values > 0.05). The characteristic time does not depend neither on the chosen geometrical parameter (AR, area, or major) nor the “process” (deformation or relaxation). Deformation and relaxation are reversible processes in terms of time. Nevertheless, the p-value corresponding to the “particle” factor (p-value < 0.05) indicates that the estimated relaxation time is different depending on the studied droplet at least in one level. In all the statistical tests, the signification coefficient is fixed at 0.05.

**Table 3:** ANOVA table using *λ*_*c*_ as response: Df are the degrees of freedom, Mean Squares are the Sum of Squares divided by the degrees of freedom, F is the value of the statistic test while p-value is the probability of obtaining a sample farthest from null hypothesis (the value of the response is the same for all the levels of the factor) than the obtained using the present sample. If p-value < 0.05, we suppose that the factor is significant with respect to response.

Factor	Df	Sum of squares	Mean squares	F value	p-value
**Magnitude**	2	21.86	10.93	1.365	0.2761
**Droplet**	4	147.3	36.83	4.601	0.0075
**Process**	1	1.780	1.78	0.0340	0.6417
**Residuals**	22	176.1	8.00		

The dependence of relaxation time on droplet was studied by implementing a multiple comparison with Tukey test [15]. Table 4 shows the results of multiple comparisons, where “diff” is the difference between the λ_c_ means of each two compared levels, “lower” is the lower end point of the 95% confidence interval for this difference, “upper” gives the upper end point, and the “adjusted p-value” is the p-value of Tukey test. Observing the p-values column, the smallest droplet 3 is different to the other ones in terms of λ_c_ (p-values < 0.05 when compared to 4, 5 and 2). It is important to note that this result is in concordance with the trends of the fits obtained in Table 1. The size of the droplet significantly affects to the relaxation time estimations. These results can also be achieved by observing the lower and upper columns: if the zero value is included in the interval, the two droplets have the same value of relaxation time λ_c_.

**Table 4:** Table of multiple comparisons obtained applying the Tukey test to the relaxation time λ_c_.

Multiple comparisons between the different levels of Droplets factor				
Comparison of pair of droplets	diff	lower	upper	Adjusted p-value
**1–2**	1.801	-3.045	6.648	0.803
**1–3**	-3.232	-8.078	1.615	0.309
**1–4**	2.719	-2.127	7.565	0.475
**1–5**	2.527	-2.319	7.373	0.545
**2–3**	-5.033	-9.879	-0.186	**0.039**
**2–4**	-0.918	-5.764	3.929	0.979
**2–5**	-0.726	-5.572	4.121	0.991
**3–4**	-5.950	-10.797	-1.104	**0.011**
**3–5**	-5.758	-10.605	-0.912	**0.015**
**4–5**	-0.192	-5.038	4.654	1.000

**Fig 10:**
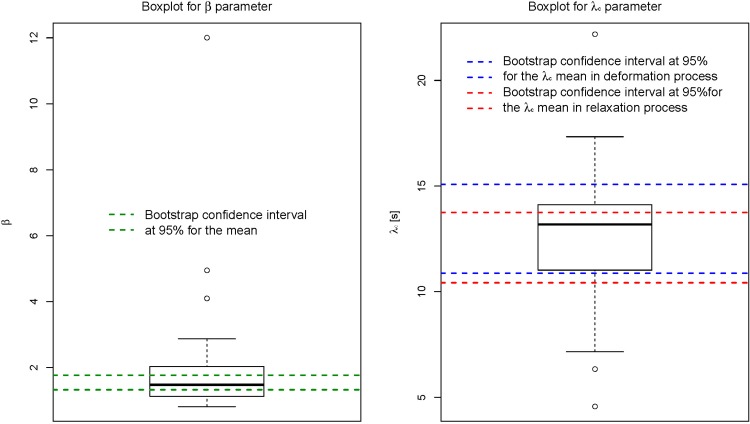
Boxplots for λ_c_ and β parameters. The line in bold represents the median, the inferior and superior edges of the box accounts for the first and third quartiles of the distribution, respectively. The points outside the exterior horizontal lines are defined as outliers.

The characteristic time of deformation/relaxation can be estimated from the KWW fits. The use of the boxplot descriptive technique gives a very informative first approximation for studying the position and dispersion of the λ_c_ parameter (Fig 10). This tool provides the median, first and third quartiles, and the extreme values that represent the limits for the distribution. The values of λ_c_ that are out of the (LI, LS) interval are suspected outliers, where LS = max{x_i_: x_i_ ≤ Q_3_ + 1.5. IQ}, LI = min{x_i_: x_i_ ≥ Q_1_−1.5. IQ}, Q_1_ is the first quartile, Q_3_ is the third quartile, and IQ is the interquartile range. We also proposed to estimate these characteristic times by using two different confidence intervals at 95% obtained by bootstrap resampling [26] without assuming any parametric assumption (as normal distribution). The confidence intervals for λ_c_ using the bootstrap R library for the deformation and relaxation are (10.87 s, 15.08 s), and (10.42 s, 13.75 s), respectively. A thousand resamples were used. The intervals are overlapped as above mentioned. This fact support that there are not significant statistical differences between the characteristic time of deformation and the characteristic time of relaxation. There are not evidences to reject the reversibility between deformation and dimension recovery in terms of relaxation time, λ_c_.

**Fig 11:**
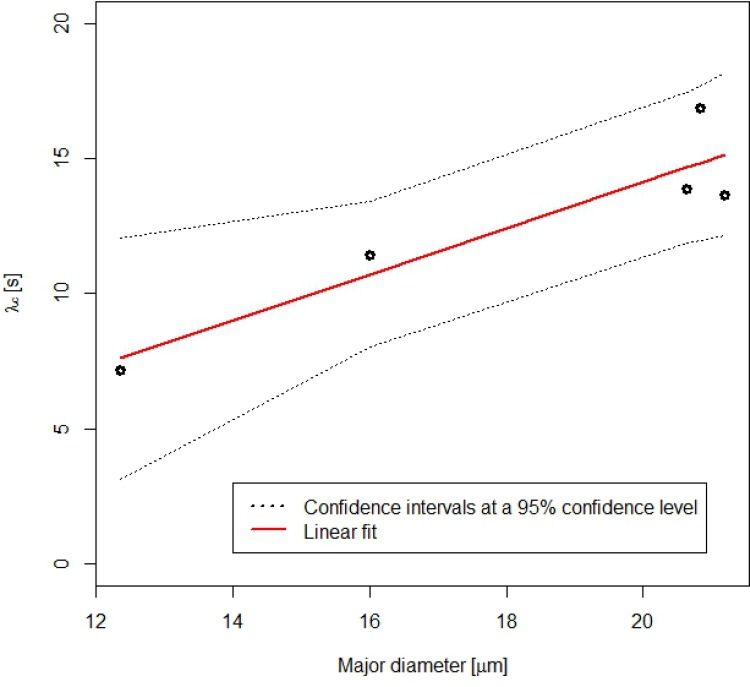
Relationship between λ_c_ and the major diameter of the droplets. Linear fit with 95% confidence levels.

It is also interesting to estimate the confidence intervals for β parameter that accounts for the number of relaxation processes in a specific material. The confidence limits at a confidence level of 95% obtained for β were (1.33, 1.77), having previously removed the outliers (Fig 9). This is in a good agreement with results obtained from KWW model (β ~ 1). Monoexponential decay was also observed for the retraction of droplet embedded in an immiscible fluid of same density after a large strain step [27]. It was explained by a reduction of the droplet-matrix interface curvature. In the present study the deformation of droplets is induced by the magnetic field. A bistability model of the shape of a concentrated magnetic droplet under applied magnetic field was developed by Bacri and Salin [28, 29]. These authors have shown that the elongation of a prolate ellipsoidal drop of magnetic permeability μ_2_ in a continuous phase of permeability μ_1_ (here assumed to be that of vacuum μ_1_~μ_0_) results from a balance between the magnetic energy E_m_ and the surface energy E_s_ which opposes the deformation. When the magnetic field is switched off, the characteristic relaxation time of a spherical droplet is λ_c_ = η_eff_ R_0_/σ where η_eff_ is the effective viscosity inside the droplet, R_0_ the radius of the spherical droplet and σ is the interfacial tension. Our experimental data are not sufficient to estimate separately η_eff_ and σ. However, the linear relationship between the λ_c_ parameter for relaxation process and the major diameter of droplets was confirmed by the application of a linear regression model to the pairs of data (see Fig 11). The estimated parametric relationship is λ_c_ = 0.852 Major diameter due to the intercept is not significant (corresponding p-value greater than any standard signification level). The effect of major diameter on the response λ_c_ is statistically significant. We have also obtained a relatively high determination coefficient, R^2^ = 0.85, that is a proof of the goodness of fit. This is in accordance with ANOVA results, where λ_c_ depends on the droplet due to the studied droplets present different sizes. The unexplained variance of the linear model and its relatively wide 95% confidence intervals are due to the uncertainty caused by the enhancement and segmentation processes of the micrographs.

## Conclusions

By using models usually applied to analyze viscoelastic properties, a new testing methodology based on dimensional analysis, image processing, Differential Evolution optimization and nonlinear modeling (Kohlrausch, Willians and Watts model) was successfully proposed to investigate magnetic field induced deformation and relaxation of droplets in an aqueous solution of sodium alginate. Geometrical changes of droplets as a function of time were obtained from the image analysis composed of image enhancement, Mixture Gaussian Modeling segmentation process and parametric regression models. Statistical techniques such as analysis of Variance (ANOVA), Multivariate Analysis of Variance (MANOVA), nonlinear regression and global optimization evolutionary algorithms were applied in order to study transition from ellipsoidal shape of droplet to spherical one as a function of time when magnetic field was switched off.

A characteristic deformation/relaxation time of the droplets in the aqueous solution of sodium alginate was estimated by using two different confidence intervals at 95% for the mean, obtained by bootstrap resampling. It was shown to not globally depend neither on the geometric parameter of the droplet (major diameter, area or ratio of major diameter by minor diameter) nor on the studied process (deformation/relaxation). Otherwise, taking into account the ANOVA results, the characteristic deformation/relaxation time depends on the studied droplet. This fact can be explained by the effect of droplet size.

The obtained values for deformation (10.87 s, 15.08 s) and relaxation (10.42 s, 13.75 s) clearly indicate a reversible magnetic field induced geometrical changes of droplets in aqueous solution of sodium alginate that could be explained by interfacial tension effects as deduced from the linear relationship between the characteristic relaxation time and the major diameter of the droplets.

Finally, the present methodology based on image segmentation and geometrical data modeling, could be applied and extended to other materials with inclusions or nanocomposites materials.
